# Targeting Autophagy Addiction in Cancer

**DOI:** 10.18632/oncotarget.384

**Published:** 2011-12-19

**Authors:** Joseph D. Mancias, Alec C. Kimmelman

**Affiliations:** ^1^ Harvard Radiation Oncology Program, Harvard Medical School, Boston, MA 02115, USA; ^2^ Division of Genomic Stability and DNA Repair, Department of Radiation Oncology, Dana-Farber Cancer Institute, Harvard Medical School, Boston, MA 02115, USA

**Keywords:** Autophagy, Ras, Metabolism, Cancer, tumor, therapy

## Abstract

Autophagy inhibition is a novel cancer therapeutic strategy in the early stages of clinical trial testing. The initial rationale for using autophagy inhibition was generated by research revealing that autophagy is upregulated in response to external stresses, including chemotherapy and radiotherapy. Combining autophagy inhibition with agents that induce autophagy as a pro-survival response may therefore increase their therapeutic efficacy. Recent research has shown that some cancer cells, particularly those driven by the K-Ras oncogene, also depend on elevated levels of autophagy for survival even in the absence of external stressors. In multiple in vitro as well as in vivo systems, oncogenic Ras-mediated transformation and tumor growth are dependent on autophagy to evade metabolic stress and cell death. These studies have subsequently led to further early phase clinical testing whether autophagy inhibition is a viable and effective strategy for targeting Ras-driven tumors. Even before the clinical results are available from these ongoing clinical trials, much work remains to optimally develop the approach of autophagy inhibition clinically; most notably reliably detecting levels of autophagy in human tumor samples, pharmacodynamics of currently available autophagy inhibitors (chloroquine and the derivative hydroxychloroquine), and new target identification and drug development.

Autophagy inhibition is at the forefront of cancer therapy, with approximately 20 ongoing clinical trials in multiple varied cancers employing this strategy as either monotherapy or in combination with other agents [1]. The initial interest in autophagy inhibition as a cancer therapy was generated by research revealing that some cancers depend on autophagy for survival during external stresses such as hypoxia, chemotherapy, or radiotherapy [2]. A new rationale for targeting autophagy has recently been elucidated by our group as well as several others showing that elevated levels of autophagy are required for cancer cells to evade lethal metabolic stress and to maintain metabolic homeostasis, particularly in tumorigenesis driven by the K-Ras oncogene [3-6]. Here we will examine the evidence for autophagy addiction as a survival strategy in Ras oncogene-mediated cancers and how current and future therapeutic strategies aim to exploit this addiction.

Macroautophagy (referred to hereafter as autophagy) is a conserved, regulated catabolic cellular pathway that degrades cellular organelles and other macromolecules [7-8]. The process involves both non-specific and targeted sequestration of cargo in a double membrane vesicle (autophagosome) that fuses to a lysosome (autolysosome), allowing for degradation of cargo and recycling of bioenergetic metabolites [9]. Autophagy has been shown to play a role in diverse disease processes including neurodegeneration, atherosclerosis, and cancer [10]. Initial research demonstrated that autophagy may function as a tumor suppressor with defects in autophagy predisposing to tumor development in certain mouse models [12]. Conversely, autophagy has also been shown to have pro-tumorigenic roles in promoting therapeutic resistance to cytotoxic chemotherapy as well as survival under stresses such as hypoxia [2]. It is likely that the function of autophagy in cancer is dynamic with both protumorigenic and tumor suppressive roles which depend on tumor stage, cellular context and tissue of origin [11]. Recently, several groups, including our own, have investigated whether autophagy plays a pro-tumorigenic role in oncogene mediated malignant transformation and subsequent tumor maintenance. Collectively the data demonstrate that in the setting of cellular transformation, oncogenic Ras expression leads to an increase in autophagy and that this upregulation is necessary for survival and for transformation. These findings suggest Ras-driven tumors may be particularly sensitive to autophagy inhibition.

Our group has examined the correlation of autophagy induction and oncogenic K-Ras both directly and indirectly [3]. While exploring the notorious treatment resistance of pancreatic ductal carcinoma (PDAC), a tumor that nearly universally possesses K-Ras mutations, we noted that all human-derived PDAC cell lines examined showed elevated basal autophagy. Autophagy was also elevated in 81% of primary PDAC tumor samples as well as in high-grade pancreatic intraepithelial neoplasms (PanINs), but was not elevated in normal pancreatic ductal epithelium or low-grade PanIN. Pharmacologic inhibition of autophagy with chloroquine (an antimalarial drug that inhibits autophagosomal degradation by interfering with lysosome pH) or genetic inhibition of autophagy in multiple PDAC cell lines attenuated growth and tumorigenicity in vitro as well as in vivo in tumor xenograft mouse models. Furthermore, chloroquine (CQ) treatment of an autochthonous K-Ras-driven genetically engineered PDAC mouse model led to a significant increase in survival as a monotherapy. This model has been shown by several groups to be highly refractory to conventional treatments as well as targeted agents [13]. Mechanistically, our work shows that autophagy is critical for proper cellular metabolism in these tumors. In particular, autophagy inhibition results in a significant decrease in oxidative phosphorylation. Together, our data provides compelling pre-clinical evidence supporting the strategy of targeting autophagy in the setting of an oncogenic K-Ras driven tumor. Coincident with our studies, several other groups have explored the relationship between oncogenic Ras-induced transformation and autophagy, further building the case for targeting autophagy addiction, not just in pancreatic cancers but in other Ras-driven tumors as well (see below).

Three recent studies have shown that autophagy is essential for oncogenic Ras-induced malignant cell transformation [4-6]. The White lab explored the functional rationale for autophagy induction in Ras-transformed cells and again demonstrates a critical metabolic function for autophagy. In a series of experiments, they show that oncogenic H-Ras and K-Ras both upregulate autophagy which supports cell survival and transformation primarily through maintenance of mitochondrial metabolic function and energy levels. The authors primarily used immortalized, non-tumorigenic baby mouse kidney epithelial cells in which they expressed oncogenic H-Ras or K-Ras to levels comparable to human cancer cell lines. Genetic inhibition of autophagy decreased cell survival during starvation and abrogated tumorigenesis in mice. The authors confirmed the relevance of their results to human cancer by showing that a number of human cancer cell lines with endogenous oncogenic Ras mutations, including pancreatic, bladder, and lung cancer cell lines, had a significant increase in the level of basal autophagy. Pharmacologic and genetic inhibition of autophagy in these cell lines revealed that a number were dependent on autophagy for cell growth, survival and proliferation. Consistent with our studies, the authors showed a decrease in oxidative phosphorylation as well a decrease in tricarboxylic acid (TCA) cycle intermediates. However, unlike in the case of our findings in pancreatic cancer, they demonstrate a buildup of damaged mitochondria due to the inhibition of mitochondrial autophagy (mitophagy). They concluded that oncogenic Ras imposes a metabolic insult on cells that depletes energy sources thereby making the cells dependent on autophagy to preserve mitochondrial function for energy production and perhaps directly provide catabolically derived metabolic substrates.

Another study explored the mechanistic link between oncogenic Ras expression and autophagy induction. Kim et al. used the spontaneously immortalized, non-transformed human breast epithelial cell line, MCF10A, and expressed constitutively active oncogenic K-Ras [5]. Elevated levels of autophagy were necessary for transformation of these cells, as pharmacologic and genetic inhibition of autophagy abrogated in vitro transformation and tumor growth in immunocompromised mice. The authors also provided evidence that oncogenic K-Ras expression upregulated autophagy via a mechanism involving ROS, p38 MAPK, and JNK activation. These conclusions warrant further exploration in complementary in vitro as well as in vivo systems, as elucidating this link could suggest additional strategies for therapeutic intervention to prevent the continued activation of autophagy in K-Ras driven tumors.

A study from the Debnath lab used oncogenic H-Ras expressing cell lines as well as several cell lines with endogenous K-Ras mutations to show that basal autophagy is elevated in oncogenic Ras expressing cells grown in non-adherent conditions, and cells deficient in autophagy exhibit decreased cell proliferation and soft agar formation [6]. Given the reliance on autophagy for cell proliferation, the authors tested whether autophagy was involved in cellular metabolism. In autophagy deficient mouse embryonic fibroblasts (MEF) expressing oncogenic Ras, there was a decrease in both glucose uptake and in glycolytic flux suggesting dependence on autophagy for glycolytic capacity. Consistent with other studies, including our own, autophagy appears to be necessary for metabolism in Ras transformed cells. However, in this case glycolysis appears to be critically affected. These differences may reflect specifics of the experimental system, such as the acute inhibition of autophagy in a chronically transformed tumor cell with CQ or RNAi versus transforming a chronically autophagy incompetent cell, such as ATG5 null MEFs. The general theme, however, is that autophagy induction supports cellular metabolism in the context of metabolic stress induced by oncogenic Ras transformation (Figure 1). The particulars with regards to cell system and tumor type deserve attention in the form of comprehensive metabolomics studies. Such studies may further elucidate targets for pharmacologic intervention.

**Figure 1:**
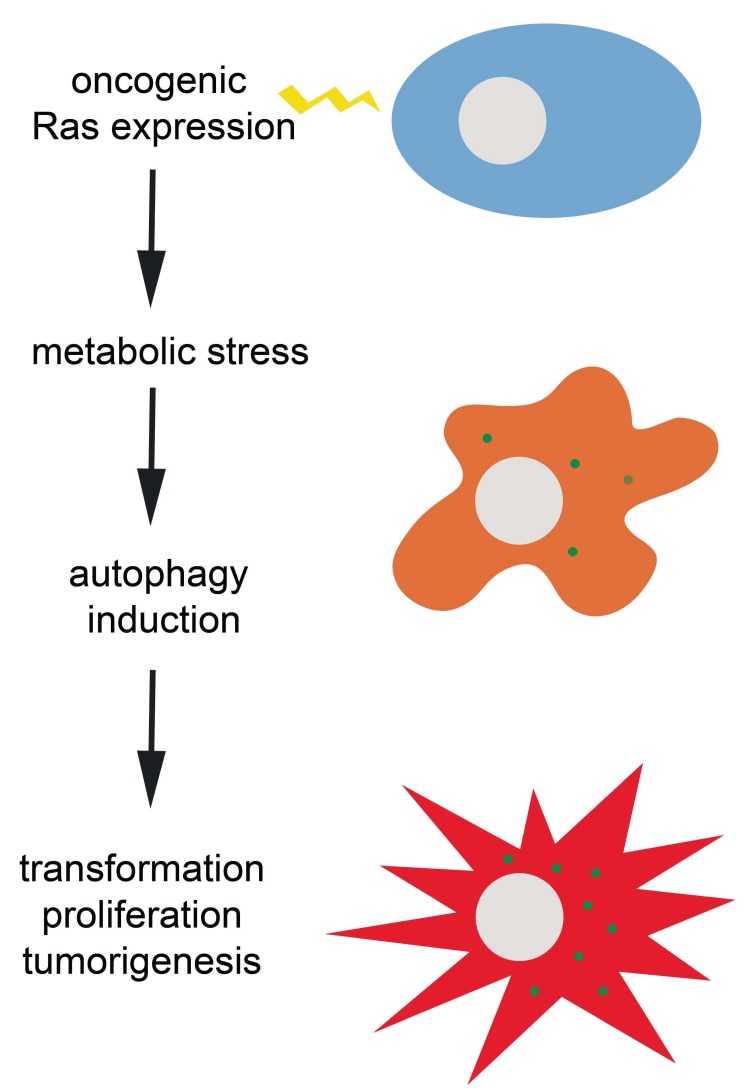
Ras oncogene-induced transformation and tumorigenesis depends on autophagy induction to evade potentially lethal metabolic stress The green dots in the orange and red cells represent autophagic puncta.

While the majority of studies have shown that oncogenic Ras results in elevated autophagy and this is critical for tumorigenesis, Elgendy et al. have shown that in some contexts oncogenic Ras expression and autophagy activation may potentially lead to cell death [14]. In their experimental system, the acute induction of oncogenic H-Ras expression causes cells to undergo an irreversible proliferative arrest and marked reduction in ability to form colonies. The authors showed that these cells have an increase in autophagic flux, but genetic inhibition of autophagy in this case decreased Ras-induced death. The authors concluded that oncogenic Ras expression leads to an autophagic cell death, which could act to limit transformation after induction of deregulated Ras signals. There are several key aspects of the study that could explain the differences with the studies that have shown that autophagy is necessary for Ras transformation. The use of a tetracycline inducible H-Ras G12V can cause an acute over-expression of H-Ras, which is in contrast to stable cell lines or endogenous mutations in tumor cell lines. Additionally, HOSE cells are not transformed by oncogenic Ras, unlike the MEFs, MCF10A, or kidney epithelial cells. Thus, while Ras activates autophagy in these cells, the ultimate cellular fate by autophagy may depend on the cellular consequence of oncogenic Ras expression. Lastly, it is important to note that the role of autophagy in promoting cellular death in mammalian systems is still somewhat controversial [15] and recent studies suggest autophagic cell death may not be a generalized phenomenon [16].

Given the pre-clinical evidence suggesting autophagy acts as a survival mechanism in response to chemotherapy, multiple clinical trials have been initiated, most studying combinations of autophagy inhibition with both traditional chemotherapy as well as targeted agents. Based on our pre-clinical evidence suggesting a pro-survival role for autophagy in pancreatic cancer [3], we have opened multiple clinical trials at our institution for patients with pancreatic cancer. The evidence presented above for the pro-survival role of autophagy in oncogenic-Ras transformed cancer cell lines suggests that other Ras-driven tumors may be particularly sensitive to autophagy inhibition as well. Additionally, the data also suggests that tumors with elevated basal autophagy will be appropriate candidates for anti-autophagy therapies as well. Determining the relevant tumor type and optimal therapeutic combinations will be critical and a significant amount of pre-clinical and clinical work is currently ongoing to answer these questions.

Even before the clinical results are available from ongoing clinical trials testing autophagy inhibition in cancer, much work remains to optimally develop the approach of autophagy inhibition clinically (Figure 2). First, our methods for measuring basal autophagy or whether autophagy is inhibited in patient samples, by IHC and western blotting for autophagic markers, are limited and of unproven utility in human patients. The pharmacodynamic studies in these ongoing clinical trials as well as in pre-clinical mouse models will be necessary to determine whether our current drugs as well as future compounds are achieving effective autophagy inhibition before we can conclusively determine the success or failure of autophagy inhibition as a therapeutic strategy. At present, all human trials exploring autophagy inhibition as a therapeutic strategy are using CQ or the derivative hydroxychloroquine given the long track record of safety in human patients. However, whether CQ and its derivatives represent the most efficacious methods for inhibiting autophagy is debatable. The high doses of CQ required to achieve tumor inhibition are obtainable in humans, but are not ideal due to the pharmacology of the drug [17-18]. Mechanistically, CQ blocks lysosome acidification, which is not specific to autophagy, but may have other potentially beneficial anti-tumor effects in addition to autophagy inhibition. There are multiple earlier steps in the autophagy pathway that may represent suitable targets, including the kinases Vps34, a class III PI3K, and ULK1/2, another family of kinases involved in the early activation of autophagy. Indeed, a recent study showed that a small molecule, Spautin-1, promoted the degradation of Vps34 by inhibiting two ubiquitin-specific proteases USP10 and USP13 that regulate the stability of the Vps34 complex [19]. By this mechanism, Spautin-1 increased cancer cell death in the setting of nutrient deprivation when autophagy would normally act as a survival mechanism in these metabolically stressed cells. This compound and strategy would require significant further pre-clinical testing before it could be employed in humans. Given the interest in autophagy inhibition, many other research groups are interested in developing improved autophagy inhibitors for cancer treatment and likewise pharmaceutical companies have also focused their attention on autophagy inhibition as a promising therapeutic avenue [20].

**Figure 2:**
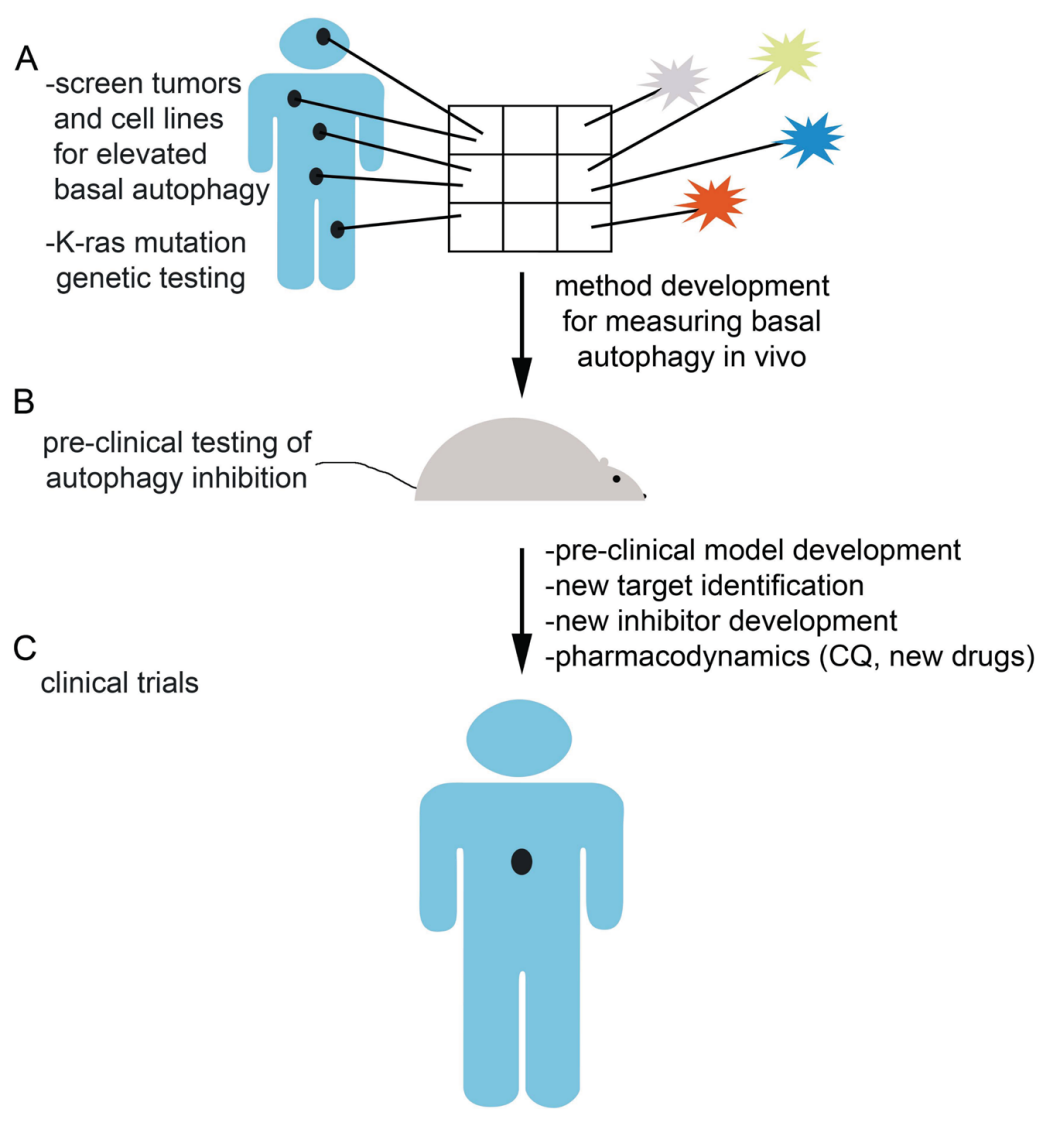
Future directions and challenges in autophagy inhibition A) Large scale screening of human tumor samples and cell lines, both oncogenic Ras-driven and otherwise, for elevated basal autophagy will identify a subset of tumors that may be particularly sensitive to autophagy inhibition as a therapeutic strategy. Method development will be required to translate this aim given the limited utility of current methods for examining human tumor samples for basal autophagy. B) Pre-clinical testing of autophagy inhibition as a therapeutic strategy in autophagy dependent tumors will lead to future clinical trials. Pre-clinical model development, pharmacodynamics of current autophagy inhibitory drugs, new target identification, and new inhibitor development are all important challenges facing the field of researchers investigating autophagy inhibition. C) Ongoing and future human clinical trials employing autophagy inhibition either as monotherapy or in combination with chemotherapy or targeted therapy will test the utility of autophagy inhibition as a cancer therapeutic strategy.

In conclusion, the studies reviewed here show that in multiple in vitro as well as in vivo systems, oncogenic Ras-mediated transformation and tumor growth are dependent on autophagy to evade metabolic stress and cell death. Autophagy inhibition is still in the early stages as a cancer therapeutic strategy but future work promises to determine whether this represents a viable and effective strategy for targeting Ras-driven tumors. While the preclinical data in mutant Ras tumors, in particular pancreatic cancers, is the most developed, there are likely to be other tumor types and driving genetic events that also exhibit autophagy addiction. Future work from multiple laboratories as well as results from ongoing clinical trials will allow us to answer these and other critical questions.
